# 1,25-Dihydroxyvitamin D_3_ Negatively Regulates the Inflammatory Response to Porcine Epidemic Diarrhea Virus Infection by Inhibiting NF-κB and JAK/STAT Signaling Pathway in IPEC-J2 Porcine Epithelial Cells

**DOI:** 10.3390/ijms231810603

**Published:** 2022-09-13

**Authors:** Jiwen Yang, Daiwen Chen, Gang Tian, Xiangbing Mao, Jun He, Ping Zheng, Jie Yu, Yuheng Luo, Junqiu Luo, Zhiqing Huang, Aimin Wu, Hui Yan, Bing Yu

**Affiliations:** Key Laboratory of Animal Disease Resistance and Nutrition, Institute of Animal Nutrition, Sichuan Agricultural University, Chengdu 611130, China

**Keywords:** 1,25(OH)_2_D_3_, inflammation, PEDV, JAK/STAT signaling pathway, NF-κB

## Abstract

Porcine epidemic diarrhea virus (PEDV) infection causes watery diarrhea and vomiting in piglets. The pathogenesis of PEDV infection is related to intestinal inflammation. It is known that 1,25-dihydroxyvitamin D_3_ (1,25(OH)_2_D_3_) has potent anti-inflammatory activity, but it is unknown whether 1,25(OH)_2_D_3_ can inhibit the PEDV-induced inflammatory response and the underlying mechanism. We used transcriptome analysis, gene and protein expression, RNA interference and overexpression, and other techniques to study the anti-inflammatory effects of 1,25(OH)_2_D_3_ on PEDV infection in IPEC-J2 cells. The results showed that interleukin 19 (*IL-19*) and C-C motif chemokine ligand 20 (*CCL20*) gene expression were enhanced with the increase in PEDV infection time in IPEC-J2 cells. Interestingly, 1,25(OH)_2_D_3_ supplementation obviously inhibited *IL-19* and *CCL20* expression induced by PEDV. Meanwhile, we also found that 1,25(OH)_2_D_3_ reduced *p*-NF-κB, *p*-STAT1, and *p*-STAT3 protein levels induced by PEDV at 24 h post-infection. IκBα and SOCS3, NF-κB, and STAT inhibitor respectively, were increased by 1,25(OH)_2_D_3_ supplementation upon PEDV infection. In addition, 1,25(OH)_2_D_3_ supplementation inhibited *ISG15* and *MxA* expression induced by PEDV. Although 1,25(OH)_2_D_3_ suppressed the JAK/STAT signal pathway and antiviral gene expression, it had no significant effects on PEDV replication and IFN-α-induced antiviral effects. In addition, when the vitamin D receptor (VDR) was silenced by siRNA, the anti-inflammatory effect of 1,25(OH)_2_D_3_ was inhibited. Meanwhile, the overexpression of VDR significantly downregulated *IL-19* and *CCL20* expression induced by PEDV infection. Together, our results provide powerful evidence that 1,25(OH)_2_D_3_ could alleviate PEDV-induced inflammation by regulating the NF-κB and JAK/STAT signaling pathways through VDR. These results suggest that vitamin D could contribute to inhibiting intestinal inflammation and alleviating intestinal damage in PEDV-infected piglets, which offers new approaches for the development of nutritional strategies to prevent PEDV infection in piglets.

## 1. Introduction

Porcine epidemic diarrhea virus (PEDV) infection causes watery diarrhea, vomiting, anorexia, and high mortality in suckling piglets [1], which leads to serious economic losses in many pig-producing countries. PEDV infects and replicates in small intestinal enterocytes and causes impaired intestinal morphology and disordered barrier function [2,3,4]. Intestinal damage caused by PEDV infection is often accompanied by increasing inflammatory cytokine expression and secretion. Wang et al. [5] reported that PEDV infection significantly enhanced *INF-α*, *IFN-β*, *TNF-α*, and *IL-6* expression in IPEC-J2 cells. In vivo, PEDV infection also stimulated proinflammatory cytokine responses [6]. In addition, NF-κB activation was found due to PEDV infection in porcine small intestinal epithelial cells [7]. PEDV not only induced an inflammatory response in porcine intestinal epithelial cells, but also increased *IL-1β*, *IL-6*, *IL-8*, and *TNF-α* expression in Vero cells [8]. This evidence indicates that PEDV infection can lead to severe inflammatory responses. The production of proinflammatory cytokines is an important part of the host innate immunity; however, an excessive rise in proinflammatory cytokines or uncontrolled inflammation would be detrimental to the intestinal structure and function. Therefore, alleviation of inflammatory cytokine expression will be conducive to the prevention of intestinal injury induced by PEDV.

It has long been thought that vitamin D (VD) not only regulates calcium and phosphorus absorption, but also plays important roles in immune regulation. Previous reports have shown that VD has broad-spectrum antiviral effects, such as against HIV [9], hepatitis C virus [10], and dengue virus [11] infection. VD also has anti-inflammatory effects, which can inhibit the inflammatory cytokine expression induced by various viral infections [11,12,13]. In addition, VD inhibits the inflammatory response to respiratory syncytial virus (RSV) infection without jeopardizing viral clearance [14]. This indicates that VD can inhibit virus-induced inflammation independently of viral clearance. Our previous study demonstrated that 25(OH)D_3_ inhibits inflammatory cytokine expression induced by PEDV infection in the jejunal mucosa of weaned pigs [15]. Therefore, it is possible that 25(OH)D_3_ can alleviate the PEDV-induced intestinal inflammatory status and injury of piglets by suppressing the proinflammatory response; however, the underlying mechanism is not clear. It is known that 1,25(OH)_2_D_3_ is the active metabolite of VD and is widely used in vitro studies. In this study, we used 1,25(OH)_2_D_3_ to explore whether it can inhibit the PEDV-induced inflammatory response and the underlying mechanism in vitro.

Increasing evidence shows that the innate immune system is always activated by viral infection, leading to NF-κB activation for interferons’ (IFNs) and other cytokines’ production [16]. Then, IFNs bind to their receptors and activate the JAK/STAT pathway to induce interferon-stimulating gene (ISG) production and establish an antiviral state [17]. However, VD can inhibit NF-κB activation and decrease the NF-κB-driven gene expression induced by RSV infection [14]. In addition, VD also can repress RSV-induced STAT1 activation and target gene expression, thereby reducing immunopathology [18]. Therefore, we postulated that VD can alleviate the PEDV-induced intestinal inflammatory status and injury by inhibiting NF-κB and JAK/STAT signaling to suppress the production of proinflammatory cytokines. 

## 2. Results

### 2.1. PEDV Induced Proinflammatory Cytokine Expression and NF-κB Activation in IPEC-J2 Cells

**Figure 1:**
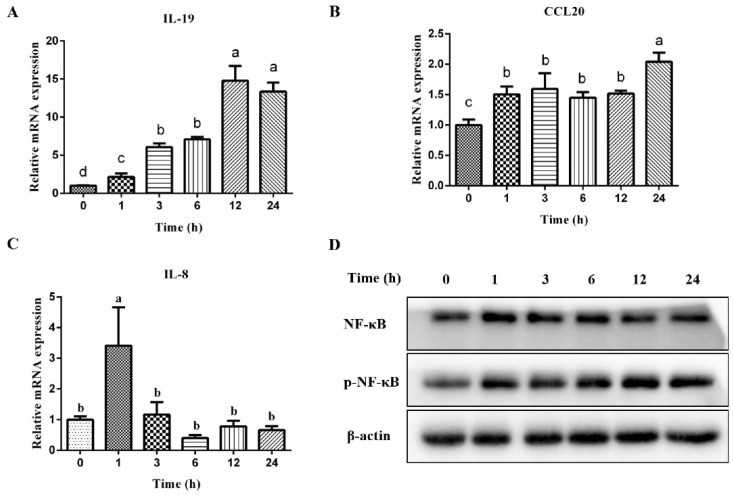
Effects of PEDV infection on inflammatory cytokine expression in IPEC-J2 cells. IPEC-J2 cells were infected with PEDV (1 MOI) for 1 h, and then the cells were washed with PBS and cultured with fresh culture medium for different times. After this, the cells were collected for RT-qPCR (**A**–**C**), n = 4 and Western blotting analysis (**D**). ^a,b,c,d^ Means not sharing the same superscript differ at *p* < 0.05.

The original differentially expressed gene data are included in the Supplementary Materials. As presented in Table 1, the transcriptomics analysis showed that PEDV infection significantly increased *IL-19* and *CCL20* mRNA expression in IPEC-J2 cells. On this basis, the mRNA expression of *IL-19* and *CCL20* was verified by real-time qPCR (RT-qPCR). PEDV infection significantly increased *IL-19* and *CCL20* gene expression with time increasing (Figure 1A,B) in IPEC-J2 cells. Moreover, we also determined *IL-8* mRNA expression with PEDV infection. Unlike the *IL-19* and *CCL20* mRNA expression profiles, PEDV only increased *IL-8* gene expression at an early time point post-infection (Figure 1C). 

NF-κB plays a central role in regulating a wide range of genes that control immunity and inflammatory responses. Thus, we proceeded to evaluate both NF-κB and phosphorylated NF-κB (*p*-NF-κB) protein levels. The results showed that PEDV infection obviously increased *p*-NF-κB levels both in the early and late stages of infection (Figure 1D).

### 2.2. 1,25(OH)_2_D_3_ Suppressed Proinflammatory Cytokine Expression and NF-κB Activation Induced by PEDV In Vitro

**Figure 2:**
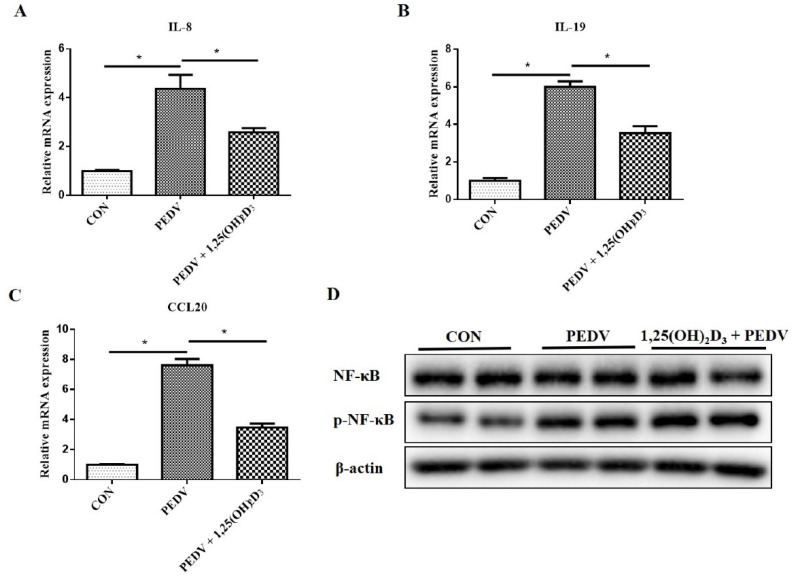
Effects of 1,25(OH)_2_D_3_ on the inflammatory cytokine expression in PEDV-infected IPEC-J2 cells at 1 h post-infection. The sub-confluent cells were incubated with 1,25(OH)_2_D_3_ (20 nM) for 24 h, after which the cells were infected with PEDV (1 MOI). After 1 h of absorption, the cells were washed with PBS and then cultured with 1,25(OH)_2_D_3_ (20 nM) for 1 h. Then, the cells were collected for RT-qPCR (**A**–**C**) and Western blotting analysis (**D**). * *p* < 0.05.

Firstly, we tested whether 1,25(OH)_2_D_3_ can inhibit the inflammatory cytokine expression induced by PEDV in IPEC-J2 cells. Results showed that pretreatment with 1,25(OH)_2_D_3_ significantly suppressed *IL-8*, *IL-19*, and *CCL20* mRNA expression induced by PEDV at 1 h post-infection (Figure 2A–C), but did not decrease *p*-NF-κB protein levels (Figure 2D).

**Figure 3:**
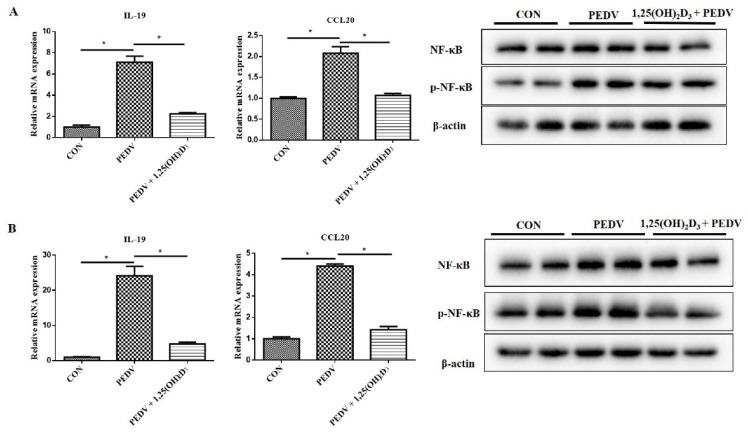
Effects of 1,25(OH)_2_D_3_ on inflammatory cytokine expression in PEDV-infected IPEC-J2 cells (**A**) and 3D4/21 cells (**B**) at 24 h post-infection. Sub-confluent cells were incubated with 1,25(OH)_2_D_3_ (20 nM) for 24 h, after which the cells were infected with PEDV (1 MOI). After 1 h of absorption, the cells were washed with PBS and cultured with 1,25(OH)_2_D_3_ (20 nM) for further 24 h. Then, the cells were collected for RT-qPCR and Western blotting analysis. * *p* < 0.05.

Nevertheless, pretreatment with 1,25(OH)_2_D_3_ significantly suppressed *IL-19* and *CCL20* mRNA expression and decreased *p*-NF-κB protein levels induced by PEDV at 24 h post-infection in IPEC-J2 cells (Figure 3A). Moreover, we also confirmed that 1,25(OH)_2_D_3_ could inhibit PEDV-induced proinflammatory cytokine expression and *p*-NF-κB protein levels at 24 h post-infection in 3D4/21 cells (Figure 3B). The results indicated that 1,25(OH)_2_D_3_ not only inhibited the inflammatory response induced by PEDV infection in intestinal epithelial cells, but also in immune cells.

**Figure 4:**
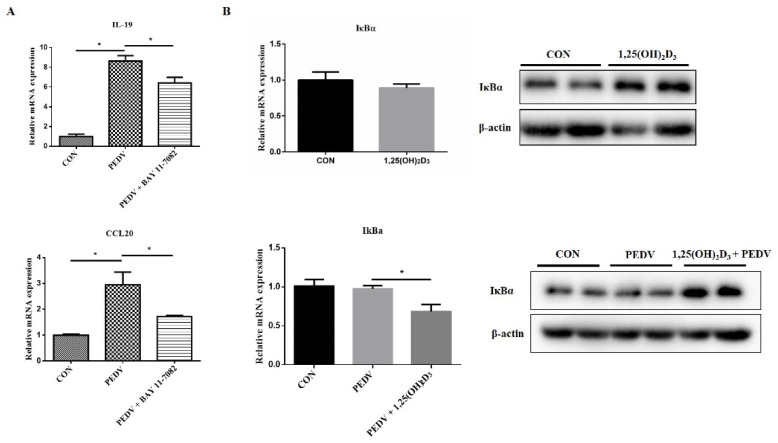
Effects of 1,25(OH)_2_D_3_ on IκBα expression in PEDV-infected IPEC-J2 cells. IPEC-J2 cells were infected with PEDV for 1 h, and then the cells were washed with PBS and incubated with BAY 11-7082 (10 μM) for 24 h. After this, the cells were collected for RT-qPCR analysis (**A**). Sub-confluent cells were incubated with 1,25(OH)_2_D_3_ (20 nM) for 24 h, and the cells were collected for RT-qPCR and Western blotting analysis or infected with PEDV (1 MOI). After 1 h of absorption, the cells were washed with PBS and then cultured with or without 1,25(OH)_2_D_3_ (20 nM) for another 24 h. Finally, the cells were collected for RT-qPCR and Western blotting analysis (**B**). * *p* < 0.05.

BAY 11-7082, an NF-κB inhibitor, was used to verify whether inhibiting NF-κB activity can suppress PEDV-induced inflammatory cytokine expression. The result showed that BAY 11-7082 decreased *IL-19* and *CCL20* mRNA expression induced by PEDV infection (Figure 4A). It suggested that 1,25(OH)_2_D_3_ could suppress PEDV-induced inflammatory cytokine expression through inhibiting NF-κB activation. In addition, we also found that IκBα, an NF-κB inhibitor, was increased by 1,25(OH)_2_D_3_ treatment in the presence or absence of PEDV infection (Figure 4B).

### 2.3. 1,25(OH)_2_D_3_ Suppressed JAK/STAT Activation Induced by PEDV in IEPC-J2 and 3D4/21 Cells

**Figure 5:**
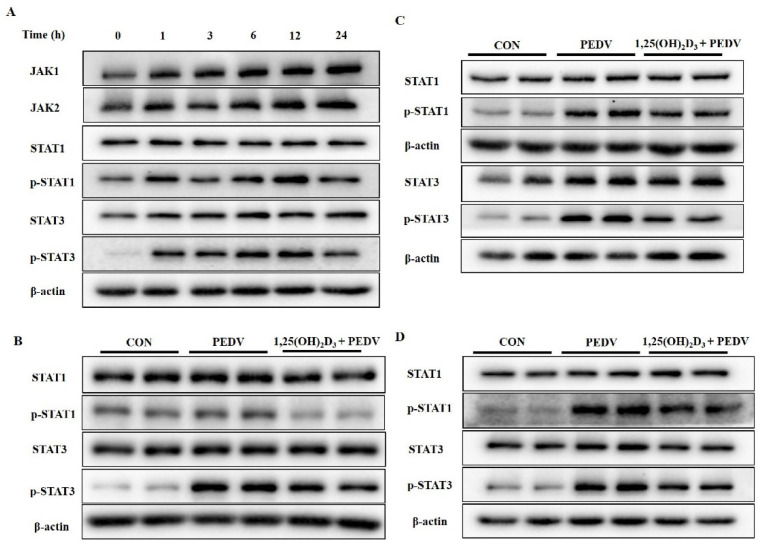
Effects of 1,25(OH)_2_D_3_ supplementation on JAK/STAT signaling pathway in PEDV-infected-IPEC-J2 and 3D4/21 cells. IPEC-J2 cells were infected with PEDV at MOI of 1. After 1 h of absorption, the cells were washed with PBS and then cultured with fresh culture medium for different times. Then, the cells were collected for Western blotting analysis (**A**). IPEC-J2 cells were pretreated with 1,25(OH)_2_D_3_ (20 nM) for 24 h, and then infected with PEDV at MOI of 1. After 1 h of absorption, the cells were washed with PBS and then cultured with 1,25(OH)_2_D_3_ (20 nM) for 1 h (**B**) or 24 h in IPEC-J2 (**C**) and 3D4/21 cells (**D**). Finally, the cells were collected for Western blotting analysis.

Since 1,25(OH)_2_D_3_ did not inhibit NF-κB activation at 1 h post-PEDV-infection, we speculated that 1,25(OH)_2_D_3_ may also inhibit the production of inflammatory cytokines through other pathways. The JAK/STAT pathway plays important roles in the regulation of immune responses and inflammatory gene expression [19,20]. We next examined JAK/STAT activation after PEDV infection. As shown in Figure 5A, PEDV infection obviously increased *p*-STAT1 and *p*-STAT3 levels in IPEC-J2 cells. However, 1,25(OH)_2_D_3_ treatment not only inhibited *p*-STAT1 and *p*-STAT3 levels at 1 h post-infection (Figure 5B), but also at 24 h post-infection (Figure 5C) in IPEC-J2 cells. Moreover, we also confirmed that 1,25(OH)_2_D_3_ could inhibit PEDV-induced JAK/STAT pathway activation at 24 h post-infection in 3D4/21 cells (Figure 5D).

**Figure 6:**
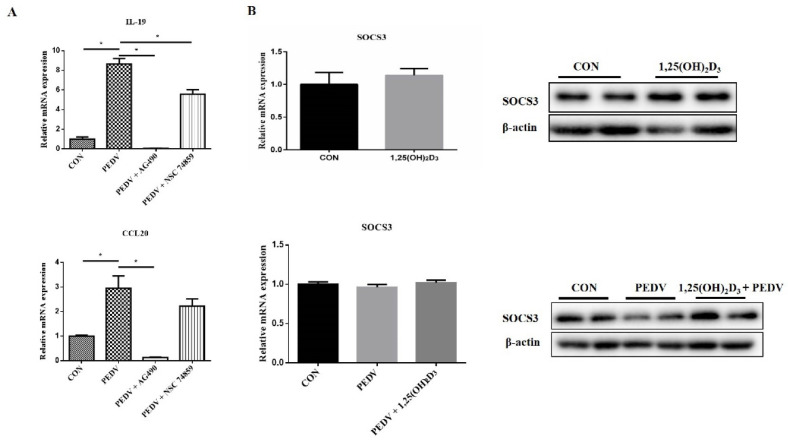
Effects of 1,25(OH)_2_D_3_ on SOCS3 expression in PEDV-infected IPEC-J2 cells. IPEC-J2 cells were infected with PEDV for 1 h, and then the cells were washed with PBS and incubated with JAK inhibitor (AG490, 100 μM) or STAT inhibitor (NSC 74859, 10 μM) for 24 h. After this, the cells were collected for RT-qPCR analysis (**A**). Sub-confluent cells were incubated with 1,25(OH)_2_D_3_ (20 nM) for 24 h, and the cells were collected for RT-qPCR and Western blotting analysis or infected with PEDV (1 MOI). After 1 h of absorption, the cells were washed with PBS and then cultured with or without 1,25(OH)_2_D_3_ (20 nM) for another 24 h. Finally, the cells were collected for RT-qPCR and Western blotting analysis (**B**). * *p* < 0.05.

In addition, a JAK inhibitor (AG490) and STAT inhibitor (NSC 74859) were used to verify whether the inhibition of the JAK/STAT pathway can inhibit the proinflammatory cytokine expression induced by PEDV infection. The results showed that both AG490 and NSC 74859 decreased the production of proinflammatory cytokines induced by PEDV (Figure 6A). This suggested that 1,25(OH)_2_D_3_ could inhibit PEDV-induced proinflammatory cytokine expression through suppressing the JAK/STAT pathway. Moreover, we also found that SOCS3, a JAK/STAT inhibitor, was increased by 1,25(OH)_2_D_3_ treatment (Figure 6B). This indicated that 1,25(OH)_2_D_3_ inhibited the JAK/STAT pathway by increasing the SOCS3 protein level, thereby inhibiting the expression of proinflammatory cytokines induced by PEDV.

### 2.4. Effects of 1,25(OH)_2_D_3_ on Antiviral Effects

**Figure 7:**
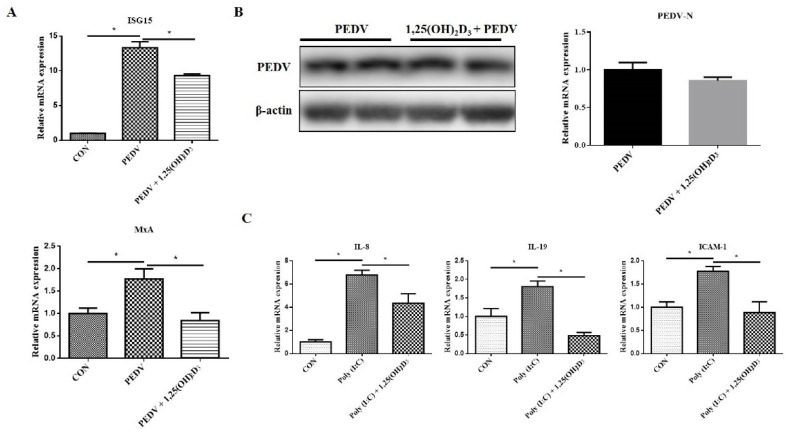
Effects of 1,25(OH)_2_D_3_ on PEDV replication. Sub-confluent cells were incubated with 1,25(OH)_2_D_3_ (20 nM) for 24 h, after which the cells were infected with PEDV. After 1 h of absorption, the cells were washed with PBS and then cultured with 1,25(OH)_2_D_3_ (20 nM) for another 24 h. Then, the cells were collected for RT-qPCR analysis (**A**) and PEDV-N mRNA and protein determination (**B**). IPEC-J2 cells were incubated with 1,25(OH)_2_D_3_ (20 nM) for 24 h, and then the cells were transfected with poly(I:C) by Lipofectamine 3000 reagent (Invitrogen) for an additional 24 h. Finally, the cells were collected for RT-qPCR analysis (**C**). * *p* < 0.05.

Generally, upon viral infection, IFNs are often produced to activate the JAK/STAT pathway, and then induce ISG expression and establish an antiviral state. Since 1,25(OH)_2_D_3_ suppressed PEDV-induced JAK/STAT activation, it is not clear whether 1,25(OH)_2_D_3_ would attenuate the ability of the IFN pathway against PEDV. As shown in Figure 7A, 1,25(OH)_2_D_3_ inhibited ISG15 and MxA expression induced by PEDV, which suggested that 1,25(OH)_2_D_3_ may be beneficial for viral replication. Fortunately, the result demonstrated that 1,25(OH)_2_D_3_ had no effect on PEDV replication after 24 h post-infection (Figure 7B), and this result is consistent with our previous study [21]. It indicated that despite the inhibition of ISG15 and MxA expression, viral replication was not influenced by 1,25(OH)_2_D_3_. In addition, we also found that 1,25(OH)_2_D_3_ inhibited poly(I:C)-induced proinflammatory cytokine expression (Figure 7C). Poly(I:C), a synthetic dsRNA, is often used to mimic viral infection. These results indicated that 1,25(OH)_2_D_3_ could inhibit the PEDV-induced excessive burst in proinflammatory cytokines but had no effect on PEDV replication after 24 h post-infection. 

**Figure 8:**
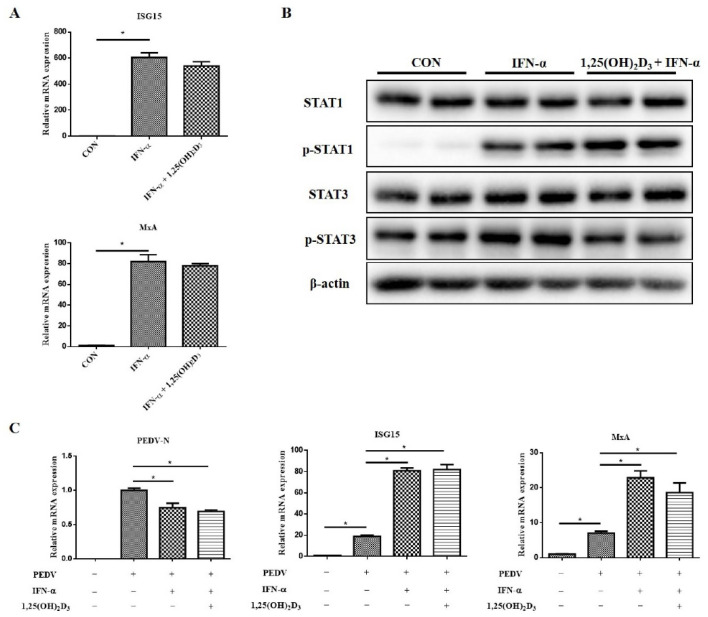
Effects of 1,25(OH)_2_D_3_ on anti-PEDV effects of IFN-α in IPEC-J2 cells. Sub-confluent cells were incubated with 1,25(OH)_2_D_3_ (20 nM) for 24 h, after which recombinant swine IFNα (1 μg/mL) was supplemented, and then the cells were cultured for further 24 h. Finally, the cells were collected for RT-qPCR analysis (**A**) and Western blotting analysis (**B**). Sub-confluent cells were incubated IFN-α (1 μg/mL) alone or with 1,25(OH)_2_D_3_ (20 nM) for 24 h, and then the cells were infected with PEDV and incubated for further 24 h. Finally, the cells were collected for RT-qPCR analysis (**C**). * *p* < 0.05.

Moreover, we examined the effects of 1,25(OH)_2_D_3_ on JAK/STAT pathway activation and antiviral gene expression with IFN-α stimulation. Here, 1,25(OH)_2_D_3_ had no effect on ISG15 and MxA expression induced by recombinant swine IFN-α (Figure 8A). We also found that 1,25(OH)_2_D_3_ decreased the IFN-α-induced *p*-STAT3 level, but it obviously increased the *p*-STAT1 level (Figure 8B). Upon PEDV infection, 1,25(OH)_2_D_3_ had no effect on *ISG15* and *MxA* expression induced by IFN-α, as well as the anti-PEDV effect of IFN-α (Figure 8C).

### 2.5. Effects of VDR on Anti-Inflammation of 1,25(OH)_2_D_3_

**Figure 9:**
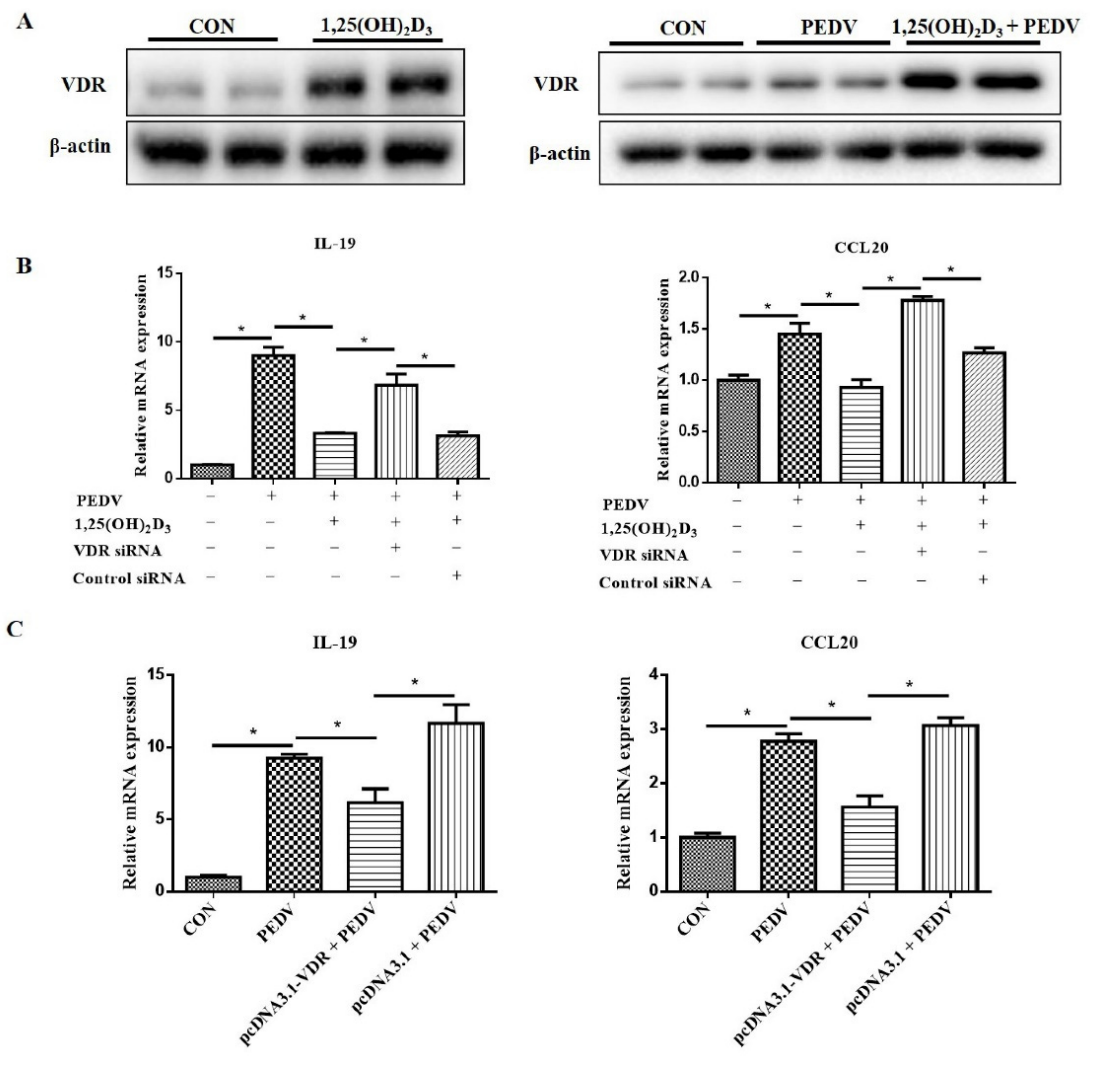
Effects of VDR on inflammatory cytokine expression in PEDV-infected IPEC-J2 cells. Sub-confluent cells were incubated with 1,25(OH)_2_D_3_ (20 nM) for 24 h, and then the cells were collected for Western blotting analysis or continued to be infected with PEDV (1 MOI). After 1 h of absorption, the cells were washed with PBS and then cultured with or without 1,25(OH)_2_D_3_ (20 nM) for further 24 h. Then, the cells were collected for Western blotting analysis (**A**). After the IPEC-J2 cells were transfected with VDR siRNA for 24 h, 1,25(OH)_2_D_3_ (20 nM) was supplemented for another 24 h, and then cells were infected with PEDV (1 MOI) and cultured for a further 24 h. Finally, the cells were harvested for RT-qPCR tests (**B**). The pcDNA3.1-VDR was transfected into IPEC-J2 cells by Lipofectamine 3000 reagent and cultured for 24 h, after which the cells were infected by PEDV and cultured for a further 24 h. Then, the cells were collected for RT-qPCR analysis (**C**). * *p* < 0.05.

Generally, 1,25(OH)_2_D_3_ exerts its functions through binding to the VD receptor (VDR). To confirm that 1,25(OH)_2_D_3_ inhibited PEDV-induced proinflammatory cytokine expression through VDR, we tested whether knockdown VDR gene expression would impact the anti-inflammatory effect of 1,25(OH)_2_D_3_. Firstly, we found that 1,25(OH)_2_D_3_ increased the VDR protein level with or without PEDV infection (Figure 9A). Then, IPEC-J2 cells were transfected with control siRNA and VDR siRNA. The results showed that the anti-inflammatory effect of 1,25(OH)_2_D_3_ was eliminated by VDR siRNA (Figure 9B). In addition, we also found that the overexpression of VDR significantly inhibited the inflammatory cytokine expression induced by PEDV (Figure 9C). 

## 3. Discussion

PEDV can infect pigs of all ages and cause high mortality in neonatal piglets, resulting in heavy economic losses in the pig breeding industry. Madson et al. [22] reported that PEDV infected intestinal epithelial cells and caused severe atrophy and variable fusion of villi with enterocyte necrosis. In addition, PEDV infection always induces a large number of inflammatory cytokines’ expression in porcine intestinal epithelial cells [5,6,7]. It is well documented that excessive intestinal inflammation can damage the integrity of the intestinal mucosal barrier, resulting in impaired intestinal barrier function [23,24]. Therefore, inhibiting PEDV-induced proinflammatory cytokines’ excessive expression is beneficial for alleviating intestinal damage. 

IL-19 plays important roles in inflammatory responses and induces apoptosis [25,26]. CCL20 is also considered to be one of the markers of inflammation [27]. In this study, the transcriptomics analysis showed that PEDV infection significantly increased *IL-19* and *CCL20* mRNA expression in IPEC-J2 cells. Increasing evidence has revealed that 1,25(OH)_2_D_3_ has a strong anti-inflammatory effect [28]. In our study, 1,25(OH)_2_D_3_ not only inhibited PEDV-induced *IL-19* and *CCL20* expression, but also downregulated poly(I:C)-induced *IL-19* and *CCL20* expression. Poly(I:C), a synthetic dsRNA, is often used to mimic viral infection. Both Drirh et al. [13] and Hansdottir et al. [14] have shown that VD inhibits the inflammatory cytokine expression induced by virus infection without affecting viral clearance. In this study, we also found that 1,25(OH)_2_D_3_ had no effect on PEDV replication. These results indicated that 1,25(OH)_2_D_3_ could inhibit the PEDV-induced proinflammatory response independently of viral clearance.

NF-κB plays a central role in regulating a wide range of genes that control immunity and inflammatory responses. NF-κB is inactive in cytoplasm through interaction with inhibitory proteins, IκBs (e.g., IκBα, IκBβ) [29]. IκBs can be phosphorylated by IκB kinase (IKK) and then degraded by the ubiquitin/proteasome pathway [30]. Afterwards, the consequence is the nuclear entry of NF-κB and this induces a variety of genes’ expression [31]. Cao et al. [6] have shown that PEDV infection induces NF-κB activation in porcine intestinal epithelial cells. However, 1,25(OH)_2_D_3_ has been found to induce IκBα expression and inhibit RSV induced-inflammatory cytokines’ expression [14]. Moreover, 1,25(OH)_2_D_3_ also decreases the DNA binding of NF-κB in keratinocyte cells [32] and 1,25(OH)_2_D_3_ exerts its functions through binding to VDR. VDR can physically interact with IKKβ and this interaction is reinforced by 1,25(OH)_2_D_3_, thereby inhibiting NF-κB activation [33]. Our results showed that 1,25(OH)_2_D_3_ treatment decreased *p*-NF-κB levels induced by PEDV and increased IκBα protein levels with or without PEDV infection. These results suggested that 1,25(OH)_2_D_3_ inhibited PEDV-induced inflammatory cytokine expression by increasing the IκBα protein level. However, in this study, we did not find that 1,25(OH)_2_D_3_ reduced the level of *p*-NF-κB at 1 h after PEDV infection. The mechanism for this phenomenon is unclear. We speculated that 1,25(OH)_2_D_3_ may induce anti-inflammatory cytokine expression at an early time post-infection, but this needs to be further investigated. 

The JAK/STAT pathway plays important roles in the regulation of immune responses and inflammatory gene expression [19,20]. Inhibition of the JAK/STAT pathway is beneficial to suppress inflammatory cytokine expression [34,35], especially reducing the levels of *p*-STAT1 and *p*-STAT3 [36,37]. In many cell types, STAT1 and STAT3 play important roles in directing cells toward cytokine responsiveness and gene expression [19]. In this study, we found that 1,25(OH)_2_D_3_ decreased *p*-STAT1 and *p*-STAT3 levels in both the early and late stage of PEDV infection. This suggested that 1,25(OH)_2_D_3_ could inhibit the inflammatory cytokine expression induced by PEDV through suppressing the JAK/STAT pathway. In addition, JAK/STAT signaling can be regulated by SOCS proteins, which directly antagonize STAT activation [19]. Previous studies suggested that 1,25(OH)_2_D_3_ can increase SOCS expression and decrease inflammatory cytokine expression [38,39]. Our results show that 1,25(OH)_2_D_3_ could increase the SOCS3 protein level with or without PEDV infection. These results indicated that 1,25(OH)_2_D_3_ suppressed the JAK/STAT signaling pathway by increasing SOCS3 expression, thereby inhibiting the inflammatory cytokine expression induced by PEDV.

Typically, the activation of the JAK/STAT pathway induced by virus infection causes ISG production [17]. ISGs are important in the host defense against viral infection. Our results demonstrated that 1,25(OH)_2_D_3_ suppressed the JAK/STAT pathway and ISG expression induced by PEDV. This raises the concern that PEDV replication may be enhanced with 1,25(OH)_2_D_3_ supplementation. Fortunately, in this study, we did not find that PEDV replication was affected by 1,25(OH)_2_D_3_ at 24 h post-infection, which is consistent with our previous study [20]. In human airway epithelial cells, 1,25(OH)_2_D_3_ also decreases RSV-induced inflammatory cytokine expression without jeopardizing viral replication [14]. One possibility is that the ISGs produced in the presence of 1,25(OH)_2_D_3_ were sufficient to restrict PEDV replication. Since 1,25(OH)_2_D_3_ inhibited the JAK/STAT antiviral signaling pathway, we sought to investigate whether 1,25(OH)_2_D_3_ could influence the antiviral effects of IFN-α. Interestingly, 1,25(OH)_2_D_3_ increased *p*-STAT1 levels and decreased *p*-STAT3 levels in the presence of IFN-α. STAT1 plays important roles in the transcription of ISGs that provide an antiviral state [40,41]. A previous study has shown that 1,25(OH)_2_D_3_ enhances the antiviral effect of IFN-α on HCV; in addition, IFN-α-induced binding of *p*-STAT1 to its DNA target sequences is also enhanced by 1,25(OH)_2_D_3_. Although, in this experiment, 1,25(OH)_2_D_3_ did not reinforce the anti-PEDV effect of IFN-α, it is further confirmed to some extent that 1,25(OH)_2_D_3_ can enhance the antiviral effect of IFN-α due to the increase in *p*-STAT1. 

It is recognized that the biological effects of 1,25(OH)_2_D_3_ are mediated by VDR [42]. In the presence of 1,25(OH)_2_D_3_, VDR heterodimerizes with retinoid X receptors (RXR). Once dimerized, the complex binds to the VDR element, in the promoter regions of 1,25(OH)_2_D_3_ target genes, to regulate their expression [43]. In addition, it is reported that 1,25(OH)_2_D_3_ can exert nongenomic actions via membrane receptor VDR to mediate cell proliferation and apoptosis [44]. There have been many reports about the relationship between VDR and inflammation. A previous study has shown that VDR^-/-^ mice developed dramatic weight loss and a colitis phenotype in TNBS and DSS colitis models, while reconstitution of VDR^-/-^ mice with the VDR transgene protected mice from developing colitis [45]. Furthermore, 1,25(OH)_2_D_3_ also inhibited the NF-κB and JAK/STAT pathways through VDR, thereby inhibiting inflammatory cytokine expression and alleviating the inflammatory response [18,33]. In this study, through RNA interference and VDR gene overexpression, we found that VDR was indispensable for 1,25(OH)_2_D_3_ to inhibit PEDV-induced inflammatory cytokine expression. However, the interaction between VDR and key proteins is not known; it is worthy of further study to elucidate the underlying molecular signaling mechanism.

**Figure 10:**
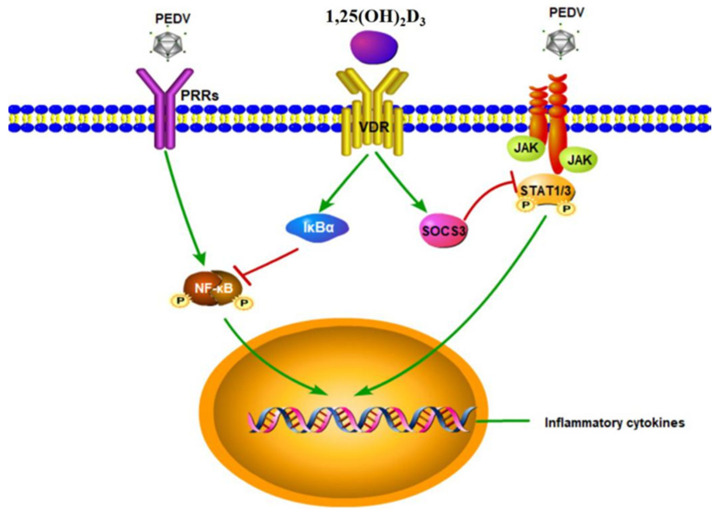
The potential mechanism of 1,25(OH)_2_D_3_ in inhibiting inflammatory cytokine expression induced by PEDV in IPEC-J2 cells. In this study, 1,25(OH)_2_D_3_ attenuated PEDV-induced inflammatory cytokine expression via suppressing the NF/κB and JAK/STAT signaling pathways.

Taken together, these results demonstrated that 1,25(OH)_2_D_3_ inhibited the proinflammatory cytokine expression induced by PEDV in IPEC-J2 cells, by inhibiting the NF-κB and JAK/STAT signaling pathways (Figure 10). These results indicated that vitamin D_3_ could contribute to inhibiting intestinal inflammation and alleviate intestinal damage in PEDV-infected piglets, offering new approaches for the development of nutritional strategies to prevent PEDV infection and reduce the risk of diarrhea in piglets. Furthermore, the results may also be helpful to guide the prevention and control of viral diarrhea in children, which is the second major cause of death by malnutrition in children under five years of age. 

## 4. Materials and Methods

### 4.1. Cells and Virus

The porcine small intestinal epithelial cell line IPEC-J2 cells were kindly provided by Per Torp Sangild (University of Copenhagen, Copenhagen, Denmark). IPEC-J2 cells were maintained in Dulbecco’s Modified Eagle’s Medium and Ham’s F-12 Nutrient Mixture (DMEM/F12, Gibco, Shanghai, China) enriched with 10% fetal bovine serum (Gibco, Shanghai, China). Porcine alveolar macrophage cell line 3D4/21 cells were grown in DMEM medium supplemented with 10% fetal bovine serum. The 3D4/21 cells were kindly provided by Professor Wenkai Ren (South China Agricultural University, Guangzhou, China). PEDV was propagated in VERO cells (African green monkey kidney cells) cultured in DMEM medium. All the cells were maintained at 37 °C in a 5% CO_2_ incubator. PEDV (the viral 50% tissue culture infectious dose (TCID_50_) of the PEDV was 5.62 × 10^7^ TCID_50_/mL) was provided by Professor Zhiwen Xu. (College of Veterinary, Sichuan Agricultural University, Chengdu, China).

### 4.2. RNA-Seq Data Analysis

After the confluent growth of IPEC-J2 cells in a 12-well plate, cells were infected with PEDV (5.62 × 10^7^ TCID_50_/mL) at an MOI of 1. After 1 h of absorption, the cells were washed with PBS and cultured for 48 h (n = 4). Then, the cells were collected. Total RNA was extracted using Trizol reagent (Invitrogen, Shanghai, China). Library construction and Illumina sequencing were conducted by the Novogene Company (Beijing, China). The fragment counts of each gene were normalized to fragments per kb of transcript per million (FPKM). Differentially expressed genes (DEGs) were screened according to |log2 Fold Change| > 0 and *p* < 0.05 by DESeq2 software. Then, the DEGs were subjected to Gene Ontology (GO) enrichment analysis by clusterProfiler software.

### 4.3. Pharmacological Inhibitors

After the confluent growth of IPEC-J2 cells (80%) in a 12-well plate, IPEC-J2 cells were infected with PEDV (5.62 × 10^7^ TCID_50_/mL) at an MOI of 1 for 1 h, and then the cells were washed with PBS and incubated with NF-κB inhibitor (BAY 11-7082, 10 μM), JAK inhibitor (AG490, 100 μM) and STAT inhibitor (WP 1066, 10 mM) for 24 h, respectively. Finally, the cells were harvested for quantitative real-time RT-PCR (RT-qPCR) tests (n = 4).

### 4.4. Plasmid Construction and Transfection

The complete CDS of VDR was synthesized by the total gene synthesis method (Shanghai Sangon Biotechnology Co., Ltd., Shanghai, China). Then, the sequence was cloned into the pcDNA3.1(+) vector, which was digested with the appropriate restriction enzyme to construct the expression vector pcDNA3.1-VDR (Shanghai Sangon Biotechnology Co., Ltd, Shanghai, China.). The pcDNA3.1-VDR was transfected into IPEC-J2 cells by Lipofectamine 3000 reagent (Invitrogen, Shanghai, China), according to the manufacturer’s instructions, and cultured for 24 h, after which the cells were infected with PEDV (5.62 × 10^7^ TCID_50_/mL, 1 MOI) and cultured for a further 24 h. Finally, the cells were collected for RT-qPCR assay. The plasmid map is included in the Supplementary Materials.

### 4.5. Poly(I:C) Transfection

When IPEC-J2 cells reached 80% confluence, the cells were incubated with 1,25(OH)_2_D_3_ (Sigma, Shanghai, China, 20 nM) for 24 h. Then, the cells were transfected with poly(I:C) (Sigma, Shanghai, China, 10 μg) by Lipofectamine 3000 reagent (Invitrogen, Shanghai, China) for an additional 24 h. Finally, the cells were collected for RT-qPCR assay.

### 4.6. IFN-α Treatment

When IPEC-J2 cells reached 80% confluence, they were pretreated with 1,25(OH)_2_D_3_ (20 nM) for 24 h, followed by recombinant swine IFN-α (Kingfisher, London, UK, 1 μg/mL) treatment for a further 24 h. Then, the cells were collected for RT-qPCR and Western blot assay. Moreover, IPEC-J2 cells were infected with PEDV (5.62 × 10^7^ TCID_50_/mL) at an MOI of 1 for 1 h, and then the cells were washed with PBS and cultured with IFN-α (1 μg/mL) and 1,25(OH)_2_D_3_ (20 nM) for 24 h. Finally, the cells were collected for RT-qPCR assay.

### 4.7. RNA Interference

The siRNA targeting porcine VDR and negative control siRNA were synthesized by Shanghai Sangon Biotechnology Co., Ltd, Shanghai, China. The sequences are listed in Table 2. IPEC-J2 cells were transfected with VDR-specific or control siRNA by Lipofectamine 3000 reagent (Invitrogen), according to the manufacturer’s instructions. After transfection for 24 h, 1,25(OH)_2_D_3_ (20 nM) was supplemented for another 24 h, and then cells were infected with PEDV (5.62 × 10^7^ TCID_50_/mL, 1 MOI) and cultured for a further 24 h. Finally, the cells were harvested for RT-qPCR test.

### 4.8. Western Blot Analysis

After washing with PBS, the cells were acquired by RIPA lysis buffer with PMSF and phosphatase inhibitor. Then, the cell samples were homogenized and centrifuged at 4 °C. The supernatants were collected. Then, the samples were separated by SDS-PAGE and transferred to PVDF membranes. The membranes were blocked by 5% nonfat milk. After this, the membranes were incubated overnight with the corresponding antibodies: anti-PEDV (Medgene Labs, Brookings, SD, USA); anti-STAT1, anti-*p*-STAT1, anti-STAT3, anti-*p*-STAT3, anti-JAK1, anti-JAK2, anti-NF-κB, anti-*p*-NF-κB, anti-IκBα (Cell Signaling Technology, Shanghai, China); anti-VDR (Abcam, Shanghai, China); anti-SOCS3 and anti-β-actin (Santa Cruz, Shanghai, China). Following washing, the samples were incubated with secondary antibodies for 1 h at room temperature, and then proteins were incubated with ECL reagent (Beyotime Biotechnology, Shanghai, China) for chemiluminescence by the ChemiDoc^TM^ XRS Imager System (Bio-Rad, Hercules, CA, USA).

### 4.9. Quantitative Real-Time RT-PCR

Total RNA was extracted using Trizol reagent (Invitrogen, Shanghai, China). The quality and concentration of RNA samples were tested by agarose gel electrophoresis and a nucleic acid analyzer (Nanodrop 2000, Thermo Scientific, Waltham, MA, USA), respectively. Then, RNA was converted into cDNA with a PrimeScript RT reagent kit (TaKaRa, Dalian, China). RT-qPCR was performed using the ABI 7900HT detection system (Applied Biosystems, Foster, CA, USA), using the SYBR Premix Ex Taq II with ROX reagents (TaKaRa, Dalian, China). The primer sequences used for RT-qPCR are listed in Table 3. The RT-qPCR reaction program was as follows: 95 °C for 30 s, then 40 cycles at 95 °C for 5 s, 60 °C for 34 s and 72 °C for 60 s. The relative mRNA expression of interested genes was quantified by the comparative ΔΔCt method by using the porcine housekeeping gene (GAPDH).

### 4.10. Statistical Analyzation

The data were presented as means ± SEM. The results of PEDV treatment for different time were analyzed by one-way analysis of variance tests followed by Tukey multiple comparison, and the other results were analyzed by Student’s *t*-test (IBM, SPSS 17.0 software, Chicago, IL, USA). The significance was declared at *p* < 0.05.

## Figures and Tables

**Table 1:** Transcription analysis results of IL-19 and CCL20 (PEDV vs. CON).

Gene ID	log2 Fold Change	*p*-Value	Padj	Gene Name
ENSSSCG00000015653	6.72	3.93 × 10^−10^	5.23 × 10^−9^	*IL-19*
ENSSSCG00000016254	1.21	8.75 × 10^−39^	1.22 × 10^−36^	*CCL20*

**Table 2:** Sequences of VDR siRNA and control siRNA.

RNA	Sense Strand Sequence (5′-3′)
VDR siRNA	Sense: CCACCGGCUUCCAUUUCAATT
	Antisense: UUGAAAUGGAAGCCGGUGGTT
Control siRNA	Sense: UUCUCCGAACGUGUCACGUTT
	Antisense: ACGUGACACGUUCGGAGAATT

**Table 3:** Primer sequences of the target and reference genes.

Gene	Primer Sequences (5′-3′)	Product Length (bp)	GeneBank Accession No.
*IL-19*	F: TCTCTGTCTCCTGGGTACGA	143	XM003130464.3
	R: GCATGGTGTCCTTAGCTTGG		
*CCL20*	F: CTGCTCTACCTCTGCAGCAA	107	NM001024589.1
	R: TGCTGTGTGAAGCCCATGAT		
*IL-8*	F: AGTTTTCCTGCTTTCTGCAGCT	72	NM_213867.1
	R: TGGCATCGAAGTTCTGCACT		
*MxA*	F: GCATCACCAGGGTAGCTGTA	195	NM_214061.2
	R: AGATCCCGATGGTCCTGTCT		
*ICAM-1*	F: GAGCTGTTCAGGCAGTCAGT	108	NM_213816.1
	R: CAGCTCAGTGCGACAAGAGA		
*IκBα*	F: TGTTGGTGTCTTTGGGTGCT	128	NM_001005150.1
	R: GACATCAGCCCCACACTTCA		
*SOCS3*	F: GAAAACAGTCAACGGCCACC	95	NM_001123196.1
	R: AAAGTGGGGCATCGTACTGG		
*ISG15*	F: TGCAAAGCTTCAGAGACCCA	145	NM_001128469.3
	R: CAGAACTGGTCAGCTTGCAC		
*PEDV-N*	F: AGATCGCCAGTTTAGCACC	66	JX_406145.1
	R: GCTCACGAACAGCCACATTA		
*GAPDH*	F: TCGCCATCAATGACCCCTTC	174	NM001206359.1
	R: CACCCCATTTGATGTTGGCG		

## Data Availability

The data presented in this study are available in the Supplementary Materials.

## Supplementary Materials

The following supporting information can be downloaded at: https://www.mdpi.com/article/10.3390/ijms231810603/s1.
