# Supplementary figures and images for: 1,25-Dihydroxyvitamin D3 Negatively Regulates the Inflammatory Response to Porcine Epidemic Diarrhea Virus Infection by Inhibiting NF-κB and JAK/STAT Signaling Pathway in IPEC-J2 Porcine Epithelial Cells
Source: Int J Mol Sci. 2022 Sep 13;23(18):10603. doi: 10.3390/ijms231810603 (PMC9504568; doi:10.3390/ijms231810603)

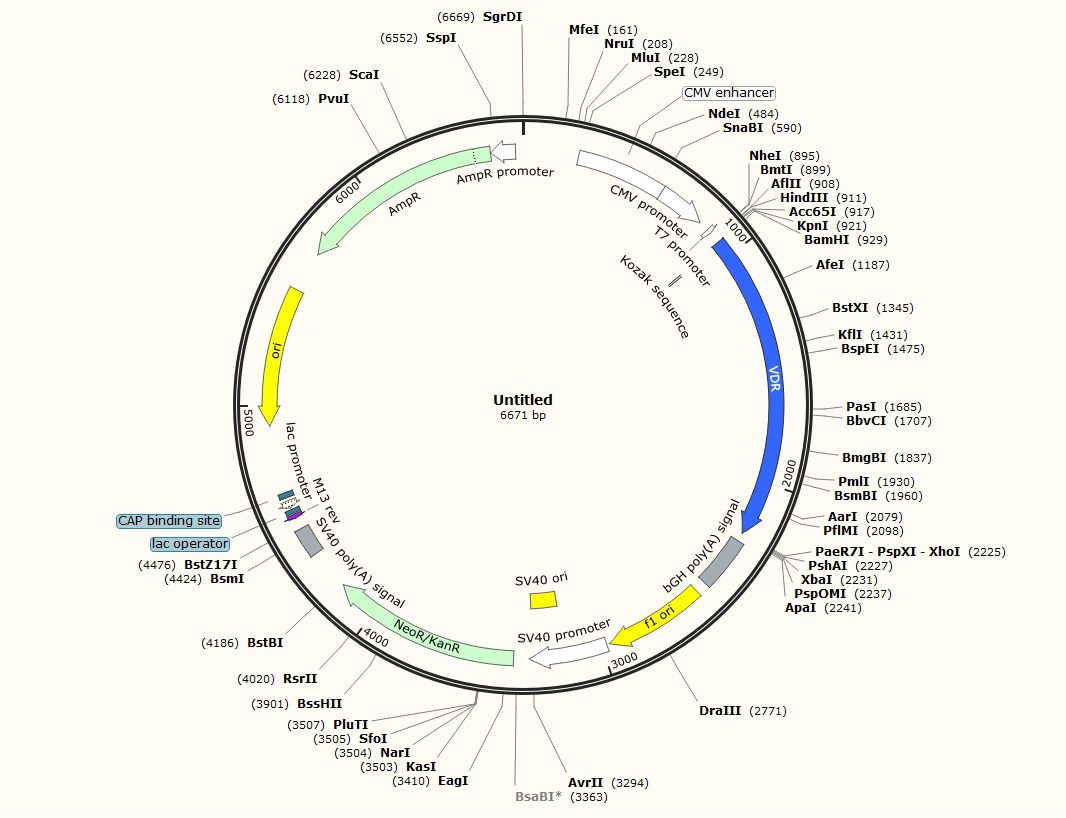

Supplement: Supplementary file 1 [file ijms-23-10603-s001.zip › Plasmid map.jpg]
